# Study on field experiments of forest soil thermoelectric power generation devices

**DOI:** 10.1371/journal.pone.0221019

**Published:** 2019-08-09

**Authors:** Yongsheng Huang, Daochun Xu, Jiangming Kan, Wenbin Li

**Affiliations:** 1 School of Technology, Beijing Forestry University, Key Lab of State Forestry Administration on Forestry Equipment and Automation, Beijing, China; 2 Institute of High Energy Physics, Chinese Academy of Sciences, Beijing, China; University of Maryland Baltimore County, UNITED STATES

## Abstract

As a new strategy to power forest wireless sensors in remote areas, an environmental microenergy collection device has been improved, and field experiments were carried out under natural conditions for the first time. The thermoelectric power generation devices used a gravity-assisted heat pipe to transmit heat from shallow soil to ground level, and a thermoelectric generator (TEG) was employed to generate electric power from the temperature difference between soil and air. Over the 6-month experimental period at two natural sites, approximately 128.74 J of energy could be harvested in a single day, and 5 209.92 J of energy could be harvested in a generation cycle. The results showed the feasibility of using this green energy to power wireless sensors in remote forests or other environments, This work is relevant to the current acute energy shortages and environmental pollution problems.

## Introduction

Primeval forests are far from human habitation; thus, it is difficult to provide electric power to forest wireless sensors because the grid is unavailable, and it is expensive to build power lines. Furthermore, batteries must be changed at regular intervals, which is labor intensive, and tall trees inhibit solar irradiation from reaching the forest floor, rendering solar energy unusable. Recently, researchers have increasingly focused on local materials, and soil is a large warehouse for energy that contains ample heat. A new method based on the Seebeck effect [1] uses the temperature difference between forest soil and air to generate electricity.

Thermoelectricity has great advantages [2], and an increasing amount of research into thermoelectricity in special environments has been conducted. Nuwayhid et al. [3,4] developed a domestic woodstove thermoelectric generator for families in rural Lebanon. They attached thermoelectric modules to domestic woodstoves and generated power with the heat released by wood burning. A single thermoelectric generator (TEG) can produce as much as 4.2 W of electric power while the stove remains available for other purposes, such as cooking and heating. Najjar and Kseibi [5] experimented further on this thermoelectrical JUST (Jordan University of Science and Technology Stove) stove with different solid fuels, demonstrating that a single TEG can generate a maximum of 5.7 W of electric power, and the maximum overall efficiency of the thermoelectric JUST stove reached 65%. A new type of flat-plate, heat-pipe-assisted TEG system was proposed for energy harvesting from hot air [6], in which researchers placed several flat heat pipes into the channels of both cold and hot air and placed thermoelectric modules between two channels. The heat transfers from the hot air to the heat pipe to the thermoelectric modules and then to the heat pipe and further to the cold air. Zhang et al. [7,8] used an all-glass, heat-tube-type vacuum solar heat collection pipe to collect solar heat and transferred the heat to the hot side of a TEG by a gravity-assisted heat pipe. Different from previous cooling methods, Zhang cooled the heat sink fitted to the cold side of the TEG by burying a vertical hollow pipe in the soil and connecting the outlet of the pipe to the heat sink, which can be cooled by cool air from the pipe in this arrangement. Other researchers have generated thermoelectric power from the exhaust of car engines [9], waste heat in municipal wastewater [10], hot flue gases in chimneys [11] and even human body heat [12,13].

Stevens [14] used the energy in soil by transferring soil heat to the ground level through a heat exchanger; two TEGs were stacked as a pair: the hot side of the first TEG faced the ground-side heat exchanger, and the cold side of the second TEG faced the air-side heat exchanger. Experimentally, the device generated electric power at an average rate of 1046 μW. Lawrence and Snyder [15] built a thermoelectric energy harvesting device to generate power by a temperature difference between the air and the shallow soil. The energy harvesting device contained a soil heat exchanger, a heat pipe, a thermoelectric microgenerator (TEMG) and an air heat exchanger. The soil heat exchanger was placed at a certain depth below the soil surface, and the air heat exchanger was exposed to air at ground level, while the heat pipe carried heat from a soil heat exchanger to the hot side of the TEMG. Then, the heat on the cold side of the TEMG was released to the surrounding air by an air heat exchanger. However, generation experiments had never been carried out on this energy harvesting device, and only a predicted electric output figure had been provided via theoretical calculations. Lawrence and Snyder studied the performance of heat sinks of different shapes and sizes; in their work, resistive heaters were placed around a soil heat exchanger, and thermocouples were adopted to measure the temperature of the soil heat exchanger and soil at certain distances. The heaters provided constant heat for the heat exchanger.

In fact, the temperature of shallow soil is higher than that of air in winter and part of the spring and autumn in the natural forest environment, and statistical information shows that the temperature at 2 m below the soil surface is approximately 10 °C higher than that at ground level in January [16]. Based on previous work [17,18], we conclude that the energy harvesting device put forwarded by Lawrence and Snyder can be further perfected to generate power with this temperature difference. Field experiments were carried out to test whether this thermoelectric power generation device could generate adequate power to supply low-power dissipation wireless sensors in forests or other remote places where conventional power is unavailable.

## Materials and methods

**Fig 1:**
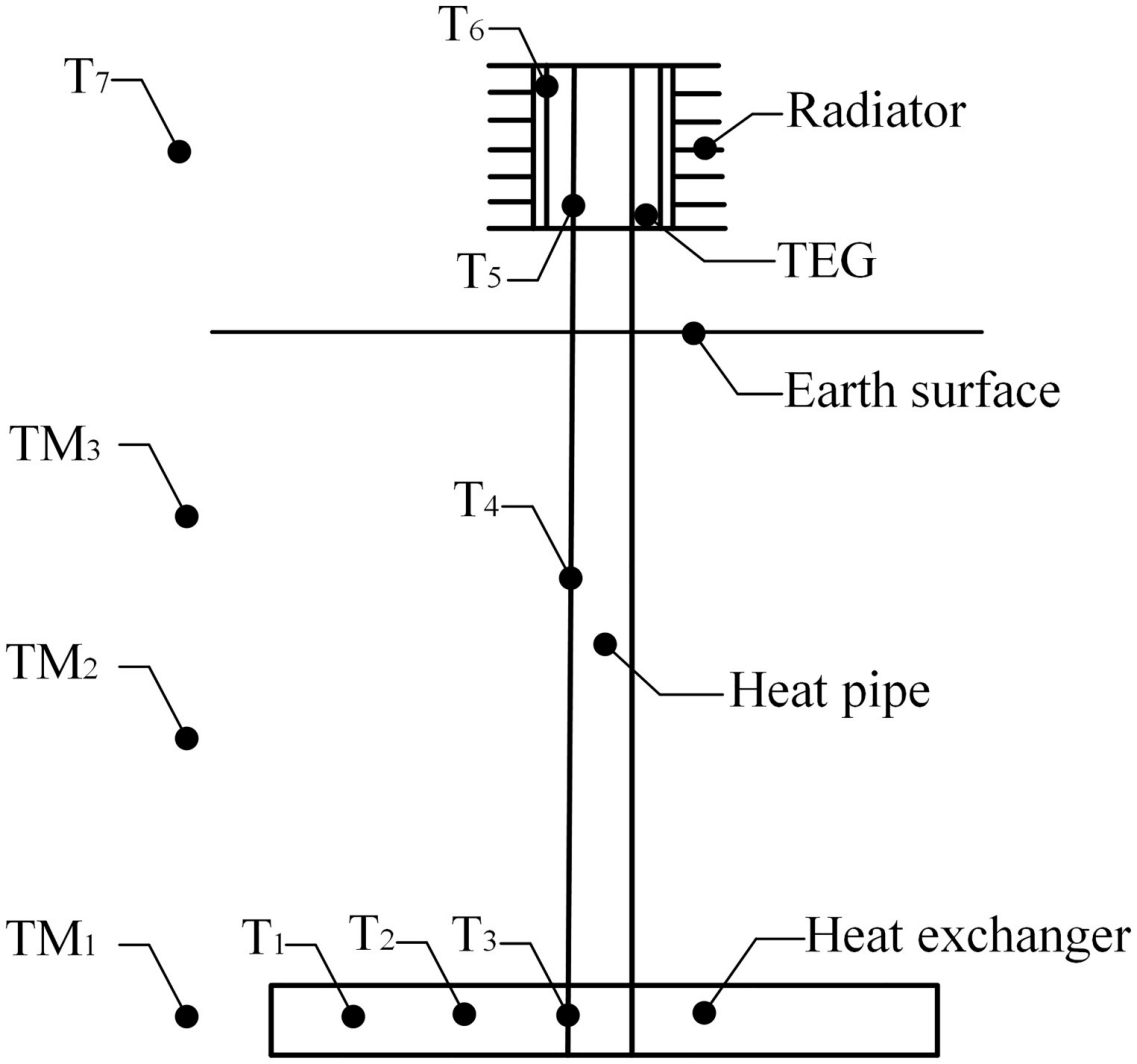
Thermoelectric power generation device. T_1_–T_7_: temperature sensors, TM_1_–TM_3_: soil temperature and moisture sensors.

As shown in Fig 1, the core elements of this thermoelectric power generation device consist of a heat exchanger, a gravity-assisted heat pipe, eight TEGs and eight radiators. The gravity-assisted heat pipe performs better at transferring heat than any other conventional metals; therefore, efficiently transmitting heat from shallow soil to ground level is feasible [19]. A gravity-assisted heat pipe is a vacuum-sealed cylinder filled with phase changeable materials, such as water, alcohol or inorganic salt. The working medium absorbs heat and evaporates in the evaporation end, and then the steam diffuses to the low-temperature condensation end, where heat is released when steam transforms back into fluid, returning to the evaporation end under the force of gravity; during this process, heat is transmitted from the soil to the hot side of the TEG. The gravity-assisted heat pipes (Custom-made, Silian Zhongke Energy Technolgy Co. Ltd, China) used in this work are 40 mm in diameter and 2.5 m or 3.5 m in length. The pipe wall is stainless steel and the working fluid is an inorganic salt mixture. One-fiftieth of the length of the pipe is the evaporation end and another one-fiftieth is the condensation end; the rest of the pipe wall is adiabatic. In practical use, the adiabatic section is wrapped with asbestos tape to reduce further heat losses. To extract more heat from the soil, a heat exchanger in the shape of a finned tube is attached to the evaporation end of the gravity-assisted heat pipe, which is a copper tube fitted with 6 flaky fins; each fin is 50 mm in width and 250 mm in length. Eight TEGs (TG12-6-02, Marlow Industries, America), 43 mm in length, 40 mm in width and 3 mm in thickness, are attached to the condensation end of the gravity-assisted heat pipe though a square outside and a cylindrical inside copper block, and the cold side is cooled by eight finned plated radiators (43 mm in length, 40 mm in width and fin height is 11 mm). Most of the thermoelectric power generation device is buried in a vertical hole, and only the condensation end of gravity-assisted heat pipe, TEGs and radiator are above ground. All contact surfaces are painted with thermally conductive silicone to improve thermal conduction.

Based on previous indoor experiments, the field tests were carried out from autumn of 2017 to spring of 2018 in Beijing City (39°54′N/116°28′E, the experiment has been authorized by Teaching & Practice Nursery of Beijing Forestry University) and Harbin City (45°14′N/126°12′E, the experiment has been permitted by rancher), Heilongjiang Province, China. In Maixner and Stevens’s work [20], the optimal depth of the device was 2.28 m at a latitude of approximately 38°N, and thus, the soil hole in Beijing was 2.3 m, and that in Harbin was 3.3 m (because the latitude is higher). The corresponding gravity-assisted heat pipe lengths were 2.5 m and 3.5 m, and the aboveground portion was 0.2 m. Because the number of TEGs was limited by the length of the condensation end of the heat pipe, eight TEGs were connected in series to increase voltage, a 1-kΩ resistor (*R*) was inserted in the circuit, and the voltage on the resistor was measured; in this way, we could reduce the error caused by the accuracy of the measuring instrument.

**Fig 2:**
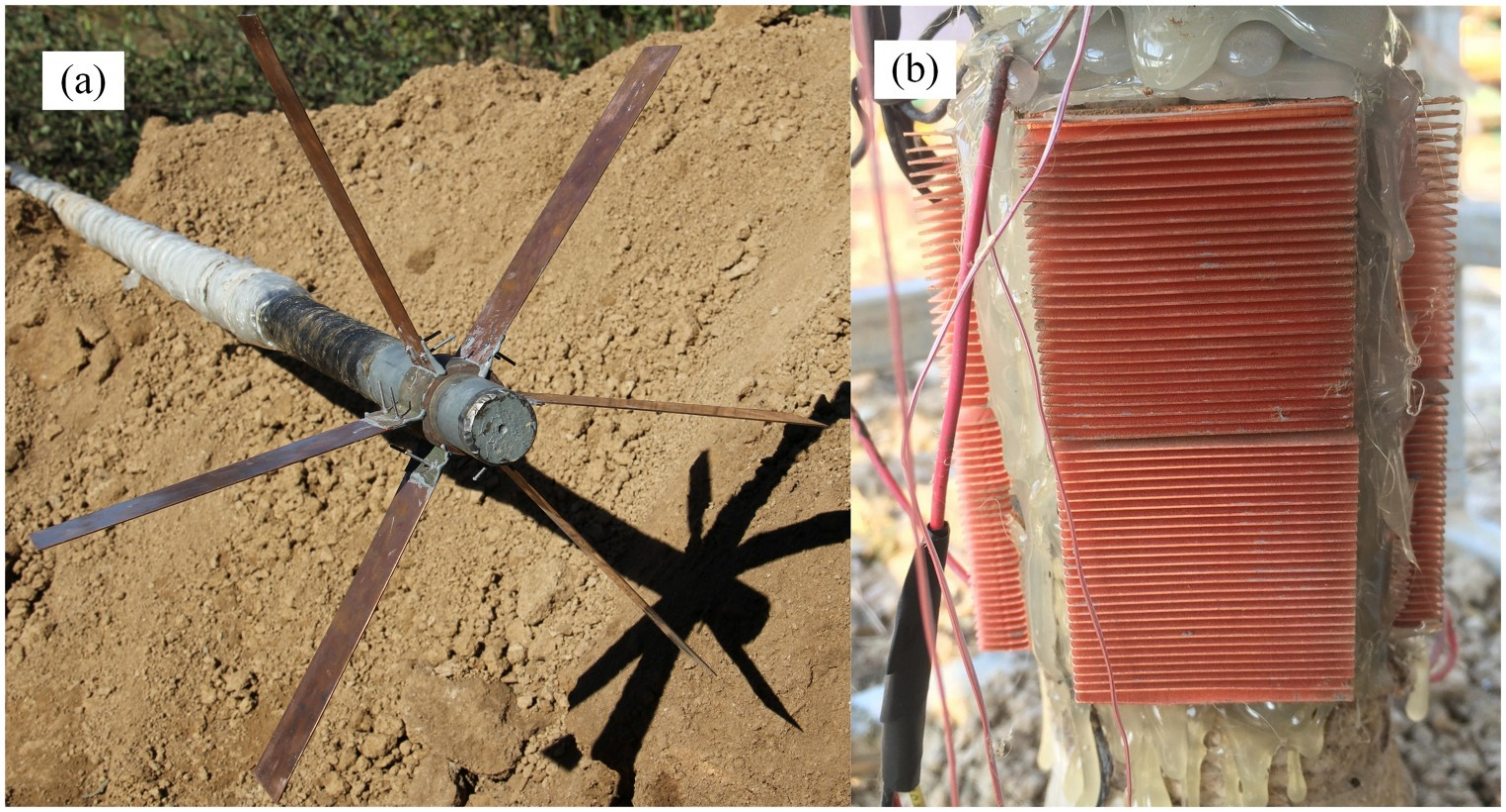
Device in field experiment. (a) Finned tube and heat pipe. (b) Radiator.

**Fig 3:**
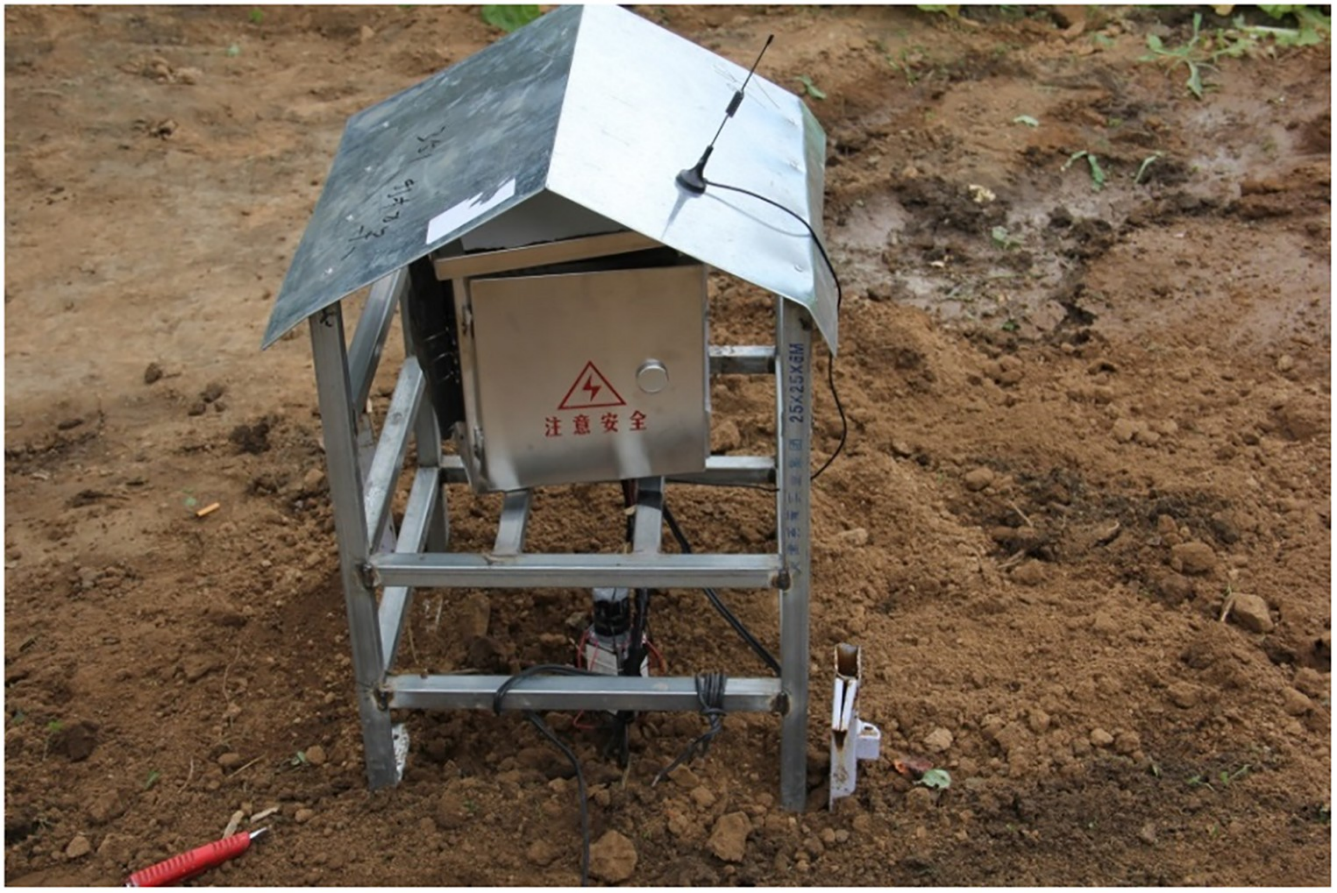
Outlook of field experiment device.

Seven thermocouples (TT-T-30-2000-SLE, Omega Engineering, America) were employed to measure the temperature on the fin (T_1_ and T_2_), the wall of the evaporation end (T_3_), the wall of adiabatic part (T_4_), the hot side of the TEG (T_5_), the cold side of the TEG (T_6_) and the air around (T_7_). The soil moisture greatly influenced thermal conduction, and three soil temperature and moisture sensors (FDS-03, Handan Qing Sheng Electronic Technology Co. Ltd, China) were placed at depths of 2.3 m, 1.3 m and 0.3 m for Beijing and 3.3 m, 2 m and 1 m for Harbin (TM_1_, TM_2_ and TM_3_) to monitor the temperature and moisture changes in the soil. All these data, including the output voltage at the resistor (*U*, measured by the data acquisition card (GPRS-1608, Toprie Electronics, China) itself), were collected by a data acquisition card and transmitted to a networking platform by a general packet radio service (GPRS). The field experiment device was showed in Figs 2 and 3.

## Results and discussion

**Fig 4:**
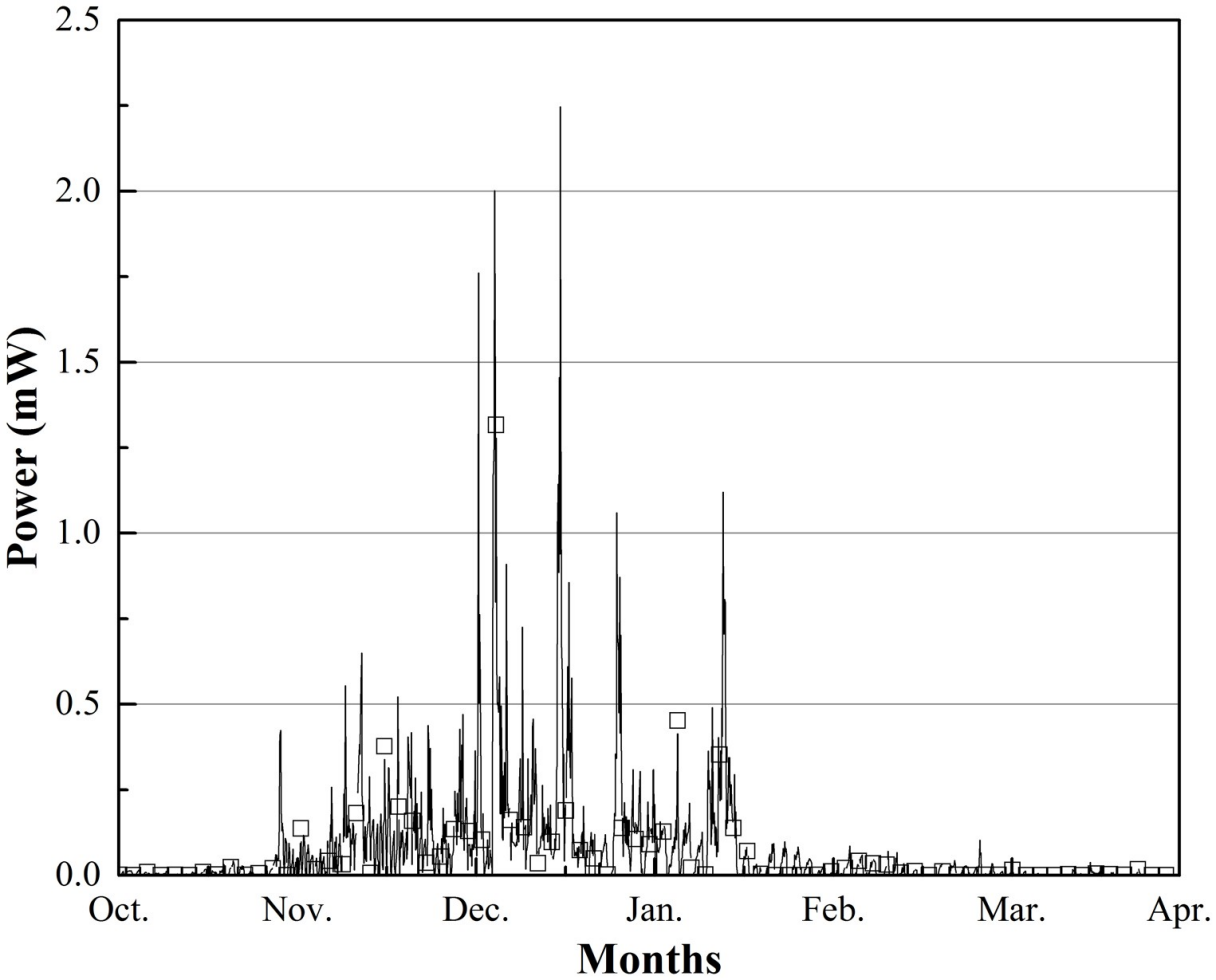
*P*_*max*_ output changes from Oct. 1 to Apr. 1 in Beijing, China.

**Fig 5:**
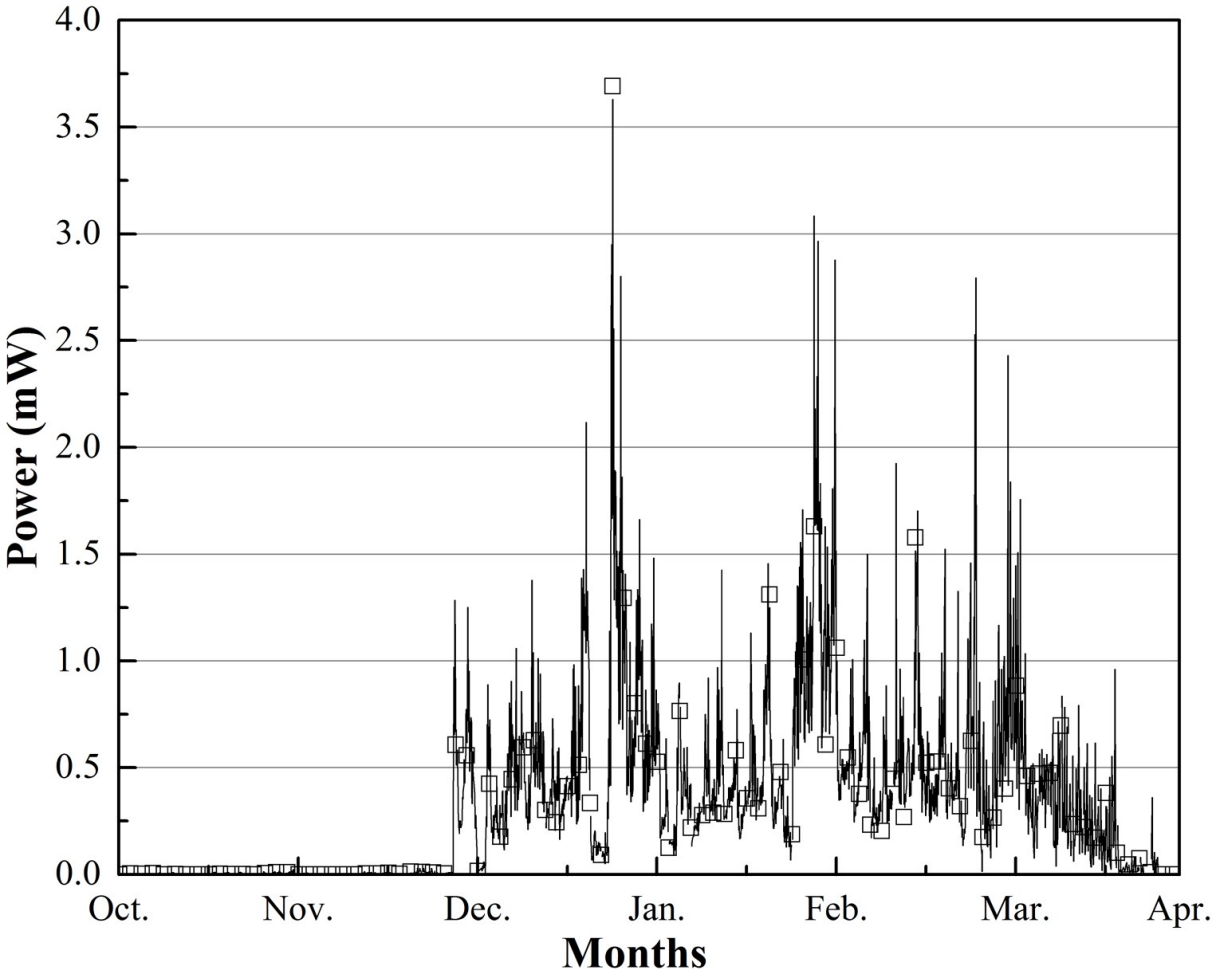
*P*_*max*_ output changes from Oct. 1 to Apr. 1 in Harbin, China.

The experiments began in Sept. 2017. Figs 4 and 5 show the changes in the calculated maximum power (*P*_*max*_) output from October 1, 2017, to April 1, 2018, when power was observed both in Beijing and Harbin. We adopted a large resistor (1 kΩ) to ensure the accuracy of output voltage measurement; thus, the electromotive force of eight TEGs was:

$$E=\frac{U}{R}(R+8\text{r})$$ (1)

where *r* was the internal resistance of one TEG, which was approximately 3.8 Ω in this project. Maximum output power was obtained if the external resistance was equal to the internal resistance of the circuit, which, in this experimental device, means that the external resistance was 8r and the output voltage was E/2. Therefore we could calculate the maximum electric power output by the following equation:

$$P_{\text{max}}=\frac{{\left(\frac{E}{2}\right)}^{2}}{8r}=\frac{{\left[\frac{\left(R+8r\right)U}{2R}\right]}^{2}}{8r}$$ (2)

All power output data are shown in Figs 4 and 5. Fig 4 shows that there was electric power output over the entire experiment time; the instability in *P*_*max*_ was typically less than 0.5 mW, and the peak value of *P*_*max*_ of approximately 2.3 mW appeared in December. In that case, the temperature difference between the hot and cold sides of the TEG was approximately 4.3 °C, and the temperature between air and soil was approximately 15.1 °C; the fastigium of power occurs from Nov. to the middle of Jan., lasting 2.5 months. The average *P*_*max*_ over the entire test was approximately 0.076 mW, and according to this rate, approximately 1 181.952 J of energy could theoretically be harvested in 6 months.

Fig 5 shows the change in *P*_*max*_ in Harbin. Compared to the value in Fig 4, this value was generally higher, the energy output was unstable, and the fastigium appeared later and lasted longer for the 4 months from December to April. The result is influenced by the climate at different sites. Harbin has a higher latitude, and the winter is colder and longer. The value of *P*_*max*_ reached a peak of 3.7 mW in December, when the temperature difference between the hot and cold sides of TEG was approximately 6.2 °C, the temperature between air and soil was approximately 26.5 °C, and the average *P*_*max*_ in Fig 5 was approximately 0.335 mW. Thus, approximately 5 209.92 J of energy could be harvested in theory.

**Fig 6:**
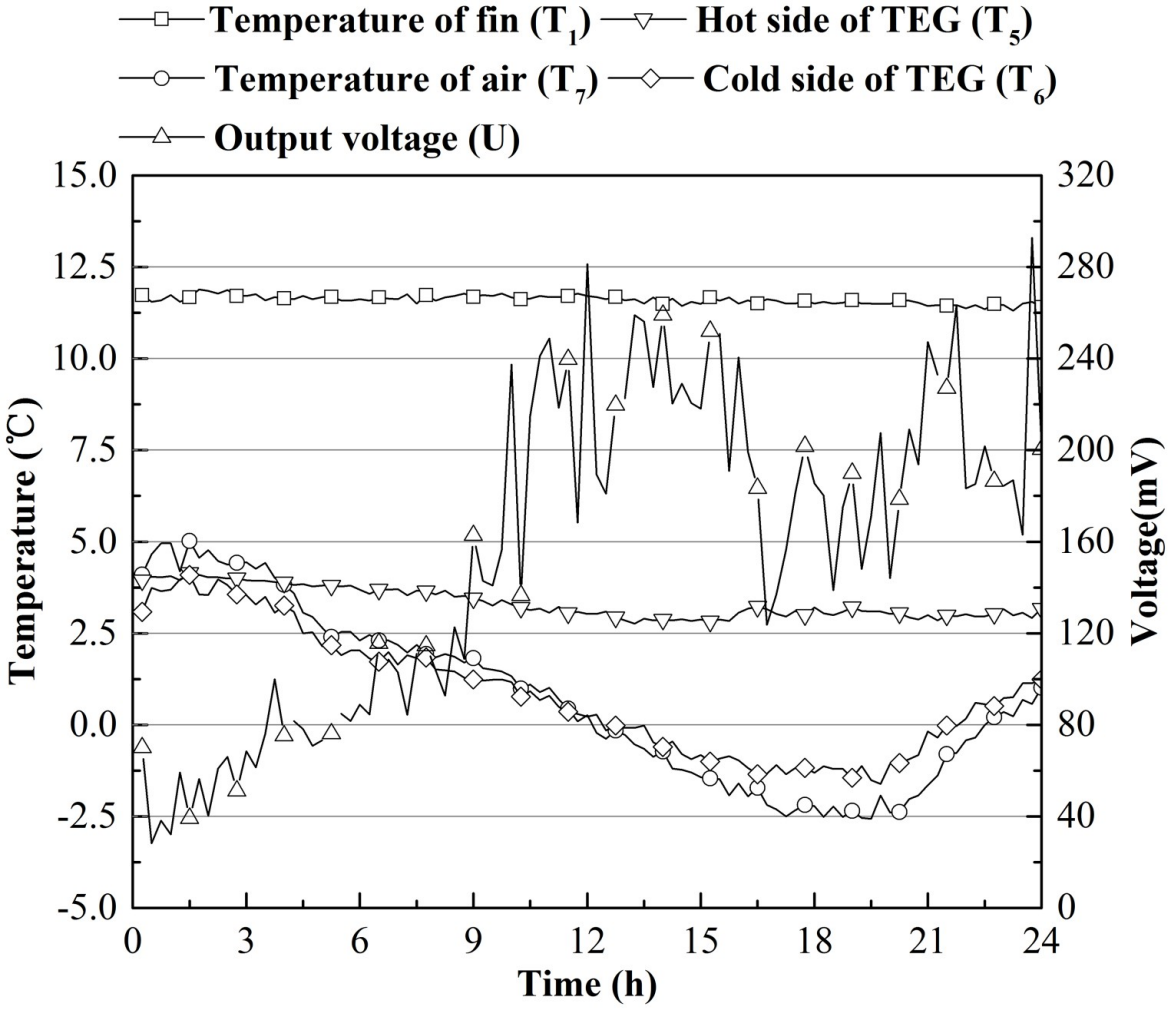
Temperature and voltage changes on the Dec. 22 in Beijing, China.

**Fig 7:**
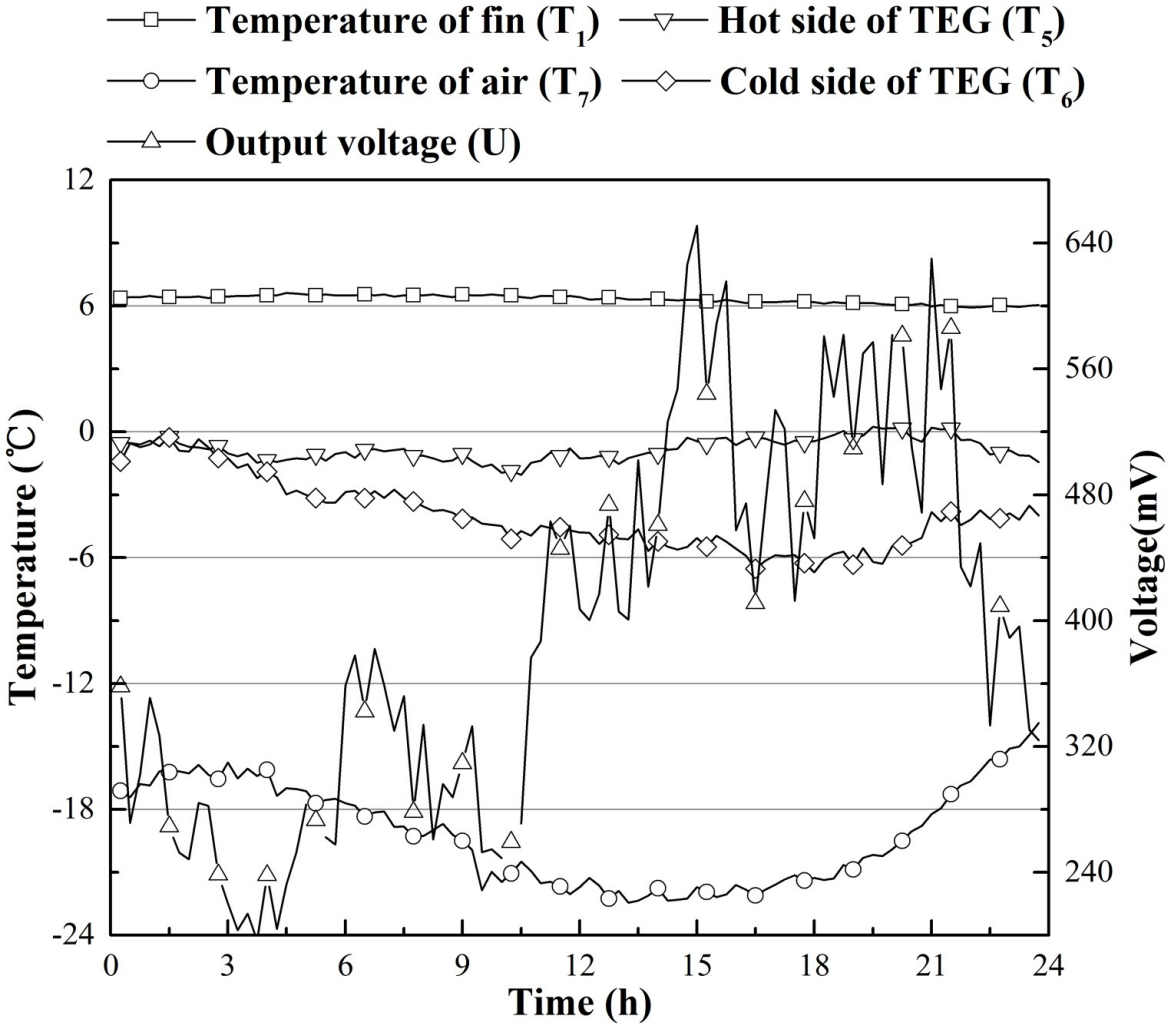
Temperature and voltage changes on the Dec. 21 in Harbin, China.

To clarify how *P*_*max*_ changed in a single day, Figs 6 and 7 were drawn to show the temperatures of the fin (T_1_), the hot side of the TEG (T_5_), the cold side of the TEG (T_6_) and the air, (T_7_) and the electric voltage output (*U*) changes over 24 hours (from 12:00, December 22, 2018 to 12:00, December 23, 2018 for Beijing, and from 12:00, December 21, 2018 to 12:00, December 22, 2018 for Harbin). Because T_1_ was on the tip of the fin, its temperature was close to that of soil. From Figs 6 and 7, the temperature of the soil beneath 2.3 m and 3.3 m does not change substantially in a single day (less than 0.5 °C), and the air temperature varied roughly as a sine wave [20]. The temperature on the hot side of the TEG fluctuated within a narrow range, while the temperature on the cold side of the TEG changed as air temperature changed. A negative correlation between the output voltage on the resistor and temperature difference on the two sides of the TEG was apparent. However, we can still see that there is a reduction in the output voltage between 15–18 hours in both Figs 6 and 7, while the temperature gap between soil and air is large. This result is due to heat constantly being released by TEGs. The rise in temperature of the air around the TEGs causes a reduction in the heat flux though the TEGs, which reduces the output voltage. This phenomenon can be solved using the wind. The voltage output in Harbin was almost double that of Beijing because there was a larger temperature difference between air and the cold side of the TEG; therefore, heat was released faster.

**Fig 8:**
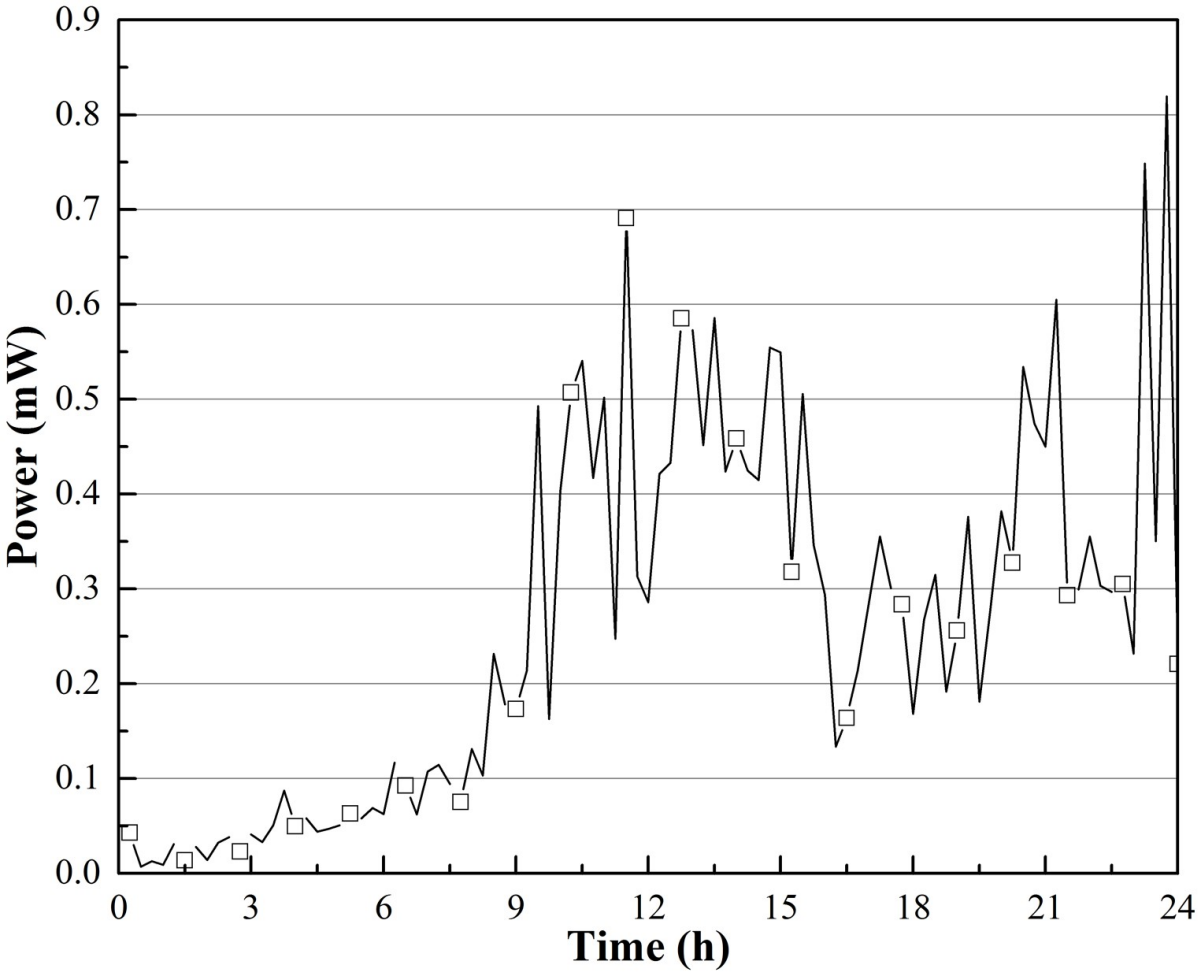
*P*_*max*_ output changes on Dec.22 in Beijing, China.

**Fig 9:**
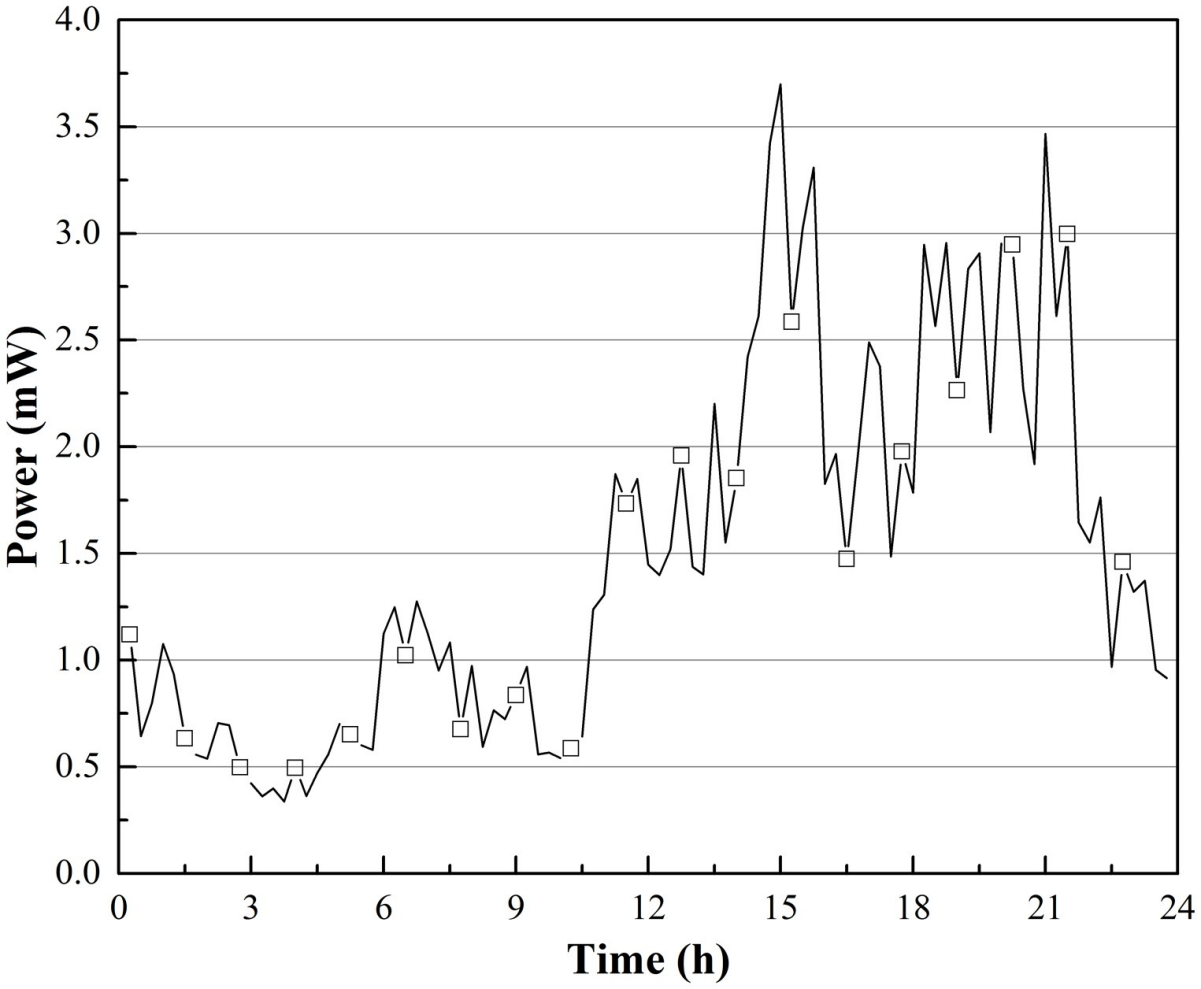
*P*_*max*_ output changes on Dec. 21 in Harbin, China.

Figs 8 and 9 show the theoretical maximum electric power output changes in the same 24 hours; the shapes of curves were the same as the corresponding voltage curves. In Fig 8, *P*_*max*_ in Beijing reached 0.82 mW (temperature difference between the hot and cold sides of the TEG was approximately 2.4 °C, and temperature difference between air and soil was approximately 10.3 °C), and the average *P*_*max*_ was approximately 0.26 mW. At this rate of work, the thermoelectric power generation device could generate approximately 22.46 J of energy in one day. *P*_*max*_ in Harbin could reach as high as 3.70 mW at approximately 3:00 in the morning, the point at which the coldest air temperature of the day occurred. *P*_*max*_ was greater than 1 mW 61.1% of the day, and the average *P*_*max*_ was approximately 1.49 mW; thus, a thermoelectric power generation device could harvest approximately 128.74 J of energy in a single day. In Lawrence and Snyder’s prototype energy harvesting device, the predicted electrical output was less than 0.4 mW, and the average value was only approximately 0.08 mW.

The electricity generated by the thermoelectric power generation devices could be boosted and then stored in a storage circuit to improve system stability. Thus, the corresponding booster and storage circuit for this device are under development. An energy system that includes other local environmental energy sources, such as solar irradiation [7,8,21], tree-based energy [22] and wind power energy [23,24], has also been developed to supplement power during summer power shortages.

Thus, Table 1 is a comparison of different thermoelectric power generation devises:

**Table 1:** Comparison of different thermoelectric power generation devises.

Researchers	Devices	Heat source	Power
Nuwayhid et al	domestic woodstove	wood burning	4.2 W
Najjar and Kseibi	JUST stove	solid fuels burning	5.7 W
Liu et al	flat-plate, heat-pipe-assisted TEG system	hot air	1.8 W
Zhang et al	solar thermoelectric power generation device	solar	362.56 mW
Orr et al	exhaust heat recovery system	engine exhaust	38 W
Zou et al	waste heat recovery system	wastewater	4.5×10^−4^ kWh m^-3^
Thielen et al	harvesting wristband	human body heat	280 μW
Stevens	ground-air thermoelectric power generator	soil	1046 μW
Huang et al	thermoelectric power generation device	soil	3.7 mW

We can examine whether the thermoelectric power generation device can power a wireless sensor in a remote area by calculation of a specific wireless sensor node. For the microprocessor MSP430, the standby current (I_s_) is 2.6 μA, and the working current (I_w_) is 500 μA. The working time (t_w_) is the sum of the sensor and the receiving and dispatching modules. The temperature sensor is TMP20, where I_s_ is 4 μA, I_w_ is 1.5 mA, and t_w_ is 1 ms. The receiving and dispatching module is nRF24l01, where I_s_ is 12 μA, I_w_ is 12.3 mA for receiving and 9 mA for dispatching, and t_w_ is 1.2 ms and 1.8 ms. In addition, there is a 1.5-ms long impulse, the current is 8.8 mA, and the node works on a voltage of 3 V. In the working module, the wireless sensor node functions in the following order: receiving data, acquisition and processing of data, and dispatching data. The energy consumption can be calculated by:

$${\text{Q}=(\text{I}}_{w}\times {\text{t}}_{w}{+\text{I}}_{s}\times {\text{t}}_{s}\text{)}\times \text{U}$$ (3)

From present data, the module consumes 0.483 J of energy in one collection and 1 738.8 J of energy in a year if it collects 10 data points per day. From the energy output at these two sites, the thermoelectric power generation device is shown to act as a wireless sensor in Harbin, and the two devices are shown to work as a wireless sensor in Beijing.

Heat transfer on the TEG is a problem of one-dimensional steady state heat conduction through a single-layer flat wall under the first boundary condition, and the heat flow (*Φ*) through eight TEGs can be calculated by the following:

$$\Phi =8\frac{\Delta T}{R_{\lambda }}$$ (4)

where *ΔT* is temperature difference between two sides of a TEG, and *R*_*λ*_ is the thermal resistance of the TEG, which is approximately 2 °C/W for outdoor temperatures. The conversion efficiency of the TEG can be obtained by dividing *Φ* into *P*_*max*_. In Harbin, the temperature difference was 4.62 °C when *P*_*max*_ reached as high as 3.70 mW. The corresponding heat flow was 18.48 W, and the conversion efficiency of the TEG was 0.020%; when *P*_*max*_ reached its highest in Beijing, 0.82 mW, the temperature difference was 1.79 °C. The heat flow though the TEGs was 7.16 W, and the conversion efficiency was 0.011%. Compared to Zou’s work [10], the energy conversion efficiency is much lower because the heat transfer capacity of soil is much lower than that of water, so the heat cannot be replenished in time. Thus far, the conversion efficiency of the thermoelectric power generation device is miniscule, and measures should be taken to enhance the thermal conductivity of soil and to improve the heat dissipation on the cold side of the TEG and the heat insulation between the upper part of the gravity-assisted heat pipe and the surrounding environment [25,26].

The thermoelectric power generation device uses the temperature difference between soil and air to function; therefore, one precondition is that the temperature of the soil is higher than that of the air. From the results above, thermoelectric power generation devices in Beijing and Harbin work only 6 months a year due to the climates at the two sites. At higher altitudes or higher latitudes, where the temperature of the soil is higher than that of the air for most of the year, the thermoelectric power generation device may function for longer. The temperature of the air around the radiator would increase due to the heat released by the radiator, and then the heat dissipation would worsen; rainfall at sites where the thermoelectric power generation devices were placed determines the moisture content in soil and affects the heat transmitted in the soil. Sunlight can also affect the temperature of the TEG; therefore, attention should be paid to these environmental factors at experimental sites in future experiments, and the influence of these factors should be analyzed.

## Conclusion

The main purpose of this paper was to determine whether the thermoelectric power generation device could produce enough electric power to run forest wireless sensors in remote areas. Two thermoelectric power generation devices were placed in the field, and data of the temperature and electric signal were monitored for 6 months. In the field experiments, the thermoelectric power generation devices used heat contained in shallow soils to generate electricity, and the highest output electric power reached 3.7 mW, with an average electric power output of 0.335 mW harvested in Harbin. Additionally, the highest electric power output reached 2.3 mW, and an average electric power output of 0.076 mW was harvested in Beijing. The results from both sites demonstrated that these devices can be used to power forest wireless sensors. By comparing those two sites, we can determine that the devices perform better in Harbin City, possibly determined by the latitude, rainfall, wind, land-sea contrast and topography, thus achieving a greater temperature difference.
